# Retraction Note: Verteporfin-induced formation of protein cross-linked oligomers and high molecular weight complexes is mediated by light and leads to cell toxicity

**DOI:** 10.1038/s41598-024-61893-8

**Published:** 2024-05-14

**Authors:** Eleni K. Konstantinou, Shoji Notomi, Cassandra Kosmidou, Katarzyna Brodowska, Ahmad Al-Moujahed, Fotini Nicolaou, Pavlina Tsoka, Evangelos Gragoudas, Joan W. Miller, Lucy H. Young, Demetrios G. Vavvas

**Affiliations:** 1 Department of Ophthalmology, Retina Service, Harvard Medical School, Boston, MA USA; 2 Pediatric Surgery Laboratories, Massachusetts General Hospital, Boston, MA 02114 USA

Retraction of: *Scientific Reports* 10.1038/srep46581, published online 21 April 2017

The Editors have retracted this Article.

After publication it was brought to the Editors’ attention that a number of the western blot images appear similar to each other, specifically:

- Figure 1B “dark (tx) & Light (lysis)” MEL 270 beta-actin appears similar to Figure 1C “dark” HEK-293 beta-actin;
- Figure 1B “dark” MCF-7 Diap1 appears similar to Figure 1C “dark” HEK-293 and MCF-7 Diap1;
- Figure 1B “dark” MEL 270 beta-actin appears similar to Figure 1B “dark” KEK 293 beta-actin;
- Figure 1C “dark (tx) and light (lysis)” 0h time point MEL 270 beta-actin appears similar to Figure 7 “MEL 270” 0h time point Diap1 beta-actin;
- Figure 1C “dark (tx) and light (lysis)” 6h time point MEL 270 Rock1 appears similar to Figure 7 “MEL 270” Diap1 6h time point;
- Figure 2 “light” Diap1 beta-actin appears similar to “dark” Diap1 beta-actin;
- Figure 2 “light” Diap1 beta-actin appears similar to Fig 3B p62 beta-actin;
- Figure 2 “light” Rock1 beta-actin appears similar to Fig 3B Rock1 beta-actin;
- Figure 2 “light” Rock1 appears similar to Figure 3A “MEL 270” Diap1 last 3 lanes;
- Figure 3A “Mel 270” p62 appears similar to Mel 270 p62 beta-actin;
- Figure 3A “MCF-7” Diap1 beta-actin appears similar to Rock1 beta-actin;
- Figure 4 “Hek 293” top beta-actin appears similar to “Hek 293” bottom beta-actin;
- Figure 4 “MCF-7” top beta-actin appears similar to “MCF-7” bottom beta-actin;
- Figure 6B “dark” p-YAP1 beta-actin appears similar to Figure 6C “light” YAP1 beta-actin;
- Figure 6B “dark” YAP1 appears similar to YAP1 beta-actin;
- Figure 6B “light” YAP1 beta-actin appears similar to Figure 6C “light” p-YAP1 beta-actin;
- Figure 6A “light” p-YAP1 appears similar to Figure 6C “light” YAP;
- Figure 6C "light" YAP1 beta-actin appears similar to Figure 7 “HEK 293” Diap1 beta-actin;
- Figure 6B "light" p-YAP1 beta-actin appears similar to Figure 7 “HEK 293” p62 beta-actin;
- Figure 7 “MEL 270” 6h time point Diap1 beta-actin appears similar to Figure 7 “HEK 293” 6h time point Rock1 beta-actin;
- Figure 7 “MCF-7” 6h time point Diap1 beta-actin appears similar to Figure 7 “MCF-7” 6h time point Rock1 beta-actin.

The Authors are unable to provide the original raw data underlying these figures. The Editors therefore no longer have confidence in the results and conclusions reported.

Eleni K. Konstantinou, Shoji Notomi, Ahmad Al-Moujahed, Fotini Nicolaou, Pavlina Tsoka, Evangelos Gragoudas, Joan W. Miller, Lucy H. Young and Demetrios Vavvas agree with this retraction and its wording. The Editors were not able to find a current email address for Cassandra Kosmidou and Katarzyna Brodowska.

